# NCOA5 Haplo-insufficiency Results in Male Mouse Infertility through Increased IL-6 Expression in the Epididymis

**DOI:** 10.1038/s41598-019-52105-9

**Published:** 2019-10-29

**Authors:** Shenglan Gao, Yueqi Zhang, Chengfeng Yang, Gloria I. Perez, Hua Xiao

**Affiliations:** 1 Department of Physiology, Michigan State University, East Lansing, Michigan 48824 USA; 2 Department of Biochemistry and Molecular Biology, Michigan State University, East Lansing, Michigan 48824 USA; 3 Cell and Molecular Biology Program, Michigan State University, East Lansing, Michigan 48824 USA; 4 Department of Radiology, Michigan State University, East Lansing, Michigan 48824 USA; 5 Present Address: Department of Cellular and Genetic Medicine, School of Basic Medical Sciences, Fudan University, Shanghai, 200032 China; 6 Present Address: Department of Toxicology and Cancer Biology, College of Medicine, University of Kentucky, Lexington, KY 40536-0305 USA

**Keywords:** Spermatogenesis, Development, Reproductive biology

## Abstract

Male infertility might be caused by genetic and/or environmental factors that impair spermatogenesis and epididymal sperm maturation. Here we report that heterozygous deletion of the nuclear receptor coactivator-5 (*Ncoa5*) gene resulted in decreased motility and progression of spermatozoa in the cauda epididymis, leading to infertility in male mice. Light microscopic and ultrastructural analysis revealed morphological defects in the spermatozoa collected from the cauda epididymis of *Ncoa5*^+/−^ mice. Immunohistochemistry showed that interleukin-6 (IL-6) expression in epithelial cells of *Ncoa5*^+/−^ epididymis was higher than wild type counterparts. Furthermore, heterozygous deletion of *Il-6* gene in *Ncoa5*^+/−^ male mice partially improved spermatozoa motility and moderately rescued infertility phenotype. Our results uncover a previously unknown physiological role of NCOA5 in the regulation of epididymal sperm maturation and suggest that NCOA5 deficiency could cause male infertility through increased IL-6 expression in epididymis.

## Introduction

Male fertility counts on effective production of mature spermatozoa that is capable of fertilizing the egg. Infertility affects about 10 to 15% couples worldwide, about 50% of which are attributed to male fertility defects, mostly due to abnormal spermatogenesis^1,2^. Spermatogenesis is a complex stepwise sequence of events in which spermatogonia develop into mature spermatozoa, also called sperm cells, through the path of mitosis and meiosis in the seminiferous tubules of the testes^3^. Spermatozoa subsequently travel to the caput and the corpus of epididymis where sperm maturation takes place. Finally, the sperm cells are stored in the cauda epididymis. After spermatogenesis, sperm are morphologically complete but immotile and unable to carry out oocyte fertilization^4^. It is only after the transit through the epididymis that the spermatozoon acquires its fertilization ability^5^, by undergoing a discrete series of post-gonadal differentiation stages controlled by the surrounding epididymal environment. Such extracellular control of gamete differentiation is called epididymal sperm maturation^6^.

The processes of spermatogenesis and sperm maturation are influenced by numerous local factors including hormone fluctuation and aberrant productions of cytokines due to metabolic syndromes and infectious diseases^5,7–9^. Accumulating evidence suggests that cytokines produced and secreted by inflammatory cells in the male urogenital tract can disturb sperm function and motility. Especially, chronic epididymitis leads to decreased sperm count and motility and is more related to male fertility than inflammation or infection of prostate and seminal vesicles^10^. Of particular interest is interleukin 6 (IL-6) that is a multifunctional cytokine involved in both pro-inflammatory and anti-inflammatory actions^11^. It is mainly expressed in immune cells including monocytes/macrophages and lymphocytes, but also expressed in many non-immune cells such as epithelial cells, astrocytes, smooth muscle cells, endothelial cells and fibroblasts in various tissues^12^. IL-6 was found to be expressed in spermatogonia, spermatocytes and interstitial cells in the testicular tissues of both immature and mature mice and its expression was decreased in testicular tissues of mature mice as compared to immature mice^13^. Human sperm cells were demonstrated to be capable of secreting IL-6. The production of IL-6 by testicular cells and its presence in seminal fluid have implicated IL-6 in the regulation of growth, differentiation and function of sperm. Indeed, IL-6 was reported to significantly reduce progressive motility at higher concentrations in a dose- and time-dependent manner^14^. IL-6 has been suggested to inhibit DNA synthesis in meiotic spermatocytes and spermatogonia within the seminiferous epithelium of rat^15^. Moreover, increased IL-6 in the semen has been reported in infertile men with varicocele^16^ and patients with infection of accessory genital glands^17^. However, little is known regarding the expression and function of IL-6 in the epididymis.

Nuclear receptor co-activator 5 (NCOA5) is a unique nuclear receptor co-activator with both co-activation and co-repression functions and regulates transcriptional activities of nuclear receptors such as estrogen receptors and liver X receptor^18–20^. Our previous study showed that NCOA5 is involved in the regulation of IL-6 transcription in liver Kupffer cells^21^. *Ncoa5*^+/−^ male mice developed glucose intolerance, hepatic steatosis and hepatocellular carcinoma (HCC), which can be partially reversed by heterozygous deletion of *Il-6* gene^21,22^. Intriguingly, we observed that *Ncoa5*^+/−^ male mice were barely able to father litters when mated with female mice, while *Ncoa5*^+/−^*Il6*^+/−^male mice could. Thus, we aimed to determine whether heterozygous deletion of *Ncoa5* affects sperm development and whether IL-6 expression mediates the effect of NCOA5 on fertility of male mice.

## Results

### Heterozygous deletion of Ncoa5 results in decreased sperm mortality and infertility in male mice

**Figure 1:**
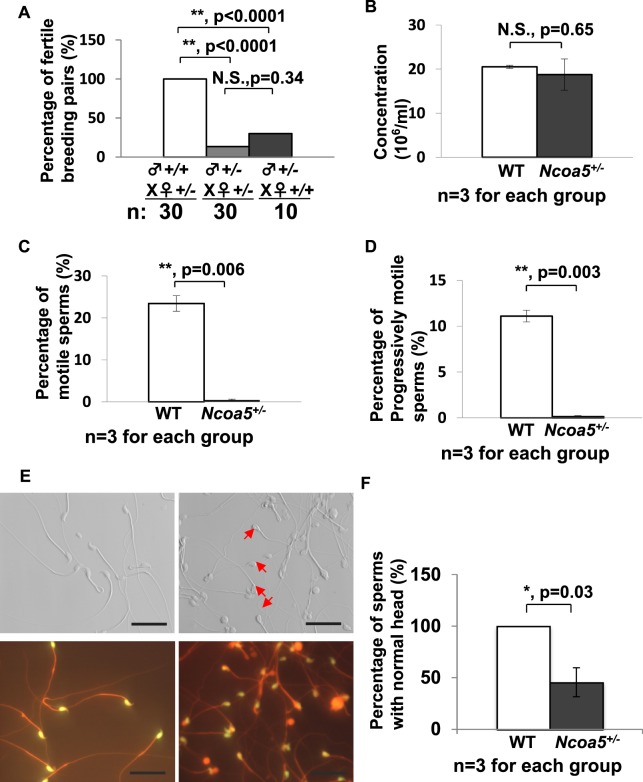
*Ncoa5*^+/−^ male mice were infertile. (**A**) Percentages of fertile WT (n = 30) or *Ncoa5*^+/−^ (n = 30) male mice bred with *Ncoa5*^+/−^ female mice and percentage of fertile *Ncoa5*^+/−^ (n = 10) male bred with WT female mice for 6 months. n = the number of mice. Two-tailed Fisher’s exact test, **P < 0.01, N.S.: no significance. (**B**) Comparison of cauda epididymis sperm concentrations between 4-month-old WT (n = 3) and *Ncoa5*^+/−^ male mice (n = 3). n = the number of mice. Values are mean ± SEM, N.S.: no significance. (**C**) Comparison of percentages of motile sperm of 4-month-old WT (n = 3) and *Ncoa5*^+/−^ male mice (n = 3). n = the number of mice. Values are mean ± SEM, **P < 0.01. (**D**) Comparison of percentages of progressive sperm of 4-month-old WT (n = 3) and *Ncoa5*^+/−^ male mice (n = 3). n = the number of mice. Values are mean ± SEM, **P < 0.01. (**E**) Phase contrast images of sperm collected from 4-month-old WT and *Ncoa5*^+/−^ mice. Red arrows indicated the abnormal head of *Ncoa5*^+/−^ male sperm. Sybr green stains the head of the sperm, while Mito track stains the mitochondria of the sperm. (**F**) Percentages of sperm with normal head in WT (n = 3) and *Ncoa5*^+/−^ male mice (n = 3). n = the number of mice. Values are mean ± SEM. *P < 0.05.

Since *Ncoa5*^−/−^ mice were not generated by breeding genetically-engineered *Ncoa5*^+/−^ male and female mice^21^, we explored whether heterozygous deletion of *Ncoa5* causes mouse infertility. According to the data in the Human Protein Atlas (www.proteinatlas.org), NCOA5 is expressed in seminiferous ducts and Leydig cells in testis, as well as in epithelial cells in the epididymis. To this end, we first compared expression of NCOA5 in the testis and epididymis between *Ncoa5*^+/+^ and *Ncoa5*^+/−^ male littermates using Western blot. As expected, we found that the protein levels of NCOA5 in *Ncoa5*^+/−^ testis were decreased (Fig. S1A,B). To determine the fertility of *Ncoa5*^+/−^ male mice, we bred a group of wild-type (WT) and *Ncoa5*^+/−^ male littermates with *Ncoa5*^+/−^ female mice over a period of 6 months. Although, there were about 13% of *Ncoa5*^+/−^ male mice (4/30) that were able to father a litter with 2–4 pups per litter at their earlier ages, the majority of *Ncoa5*^+/−^ male (26/30) mice at ages of 2–8 months were not able to father a litter, whereas all *Ncoa5*^*+/+*^ male mice were able to father multiple litters with 5–8 pups per litter (Fig. 1A). Even when bred with wild type female mice, just about 30% of *Ncoa5*^+/−^ male mice (3/10) were capable of fathering a litter (Fig. 1A). In contrast to *Ncoa5*^+/−^ males, *Ncoa5*^+/−^ females are fertile, as they were used for maintaining the *Ncoa5*^+/−^ breeding colony. This result indicates that the fertility of *Ncoa5*^+/−^ male mice is severely impaired. Analysis of sperm isolated from cauda epididymis showed that the concentration of sperm in WT and *Ncoa5*^+/−^ males is comparable (Fig. 1B). However, the percentage of motile sperm, as well as the percentage of progressively motile sperm, are significantly decreased in *Ncoa5*^+/−^ male mice compared to age-matched WT males (Fig. 1C,D). Taken together, these results suggest that *Ncoa5*^+/−^ male mice are infertile, at least in part, due to a decrease in the number of motile sperm.

### *Ncoa5*^+/−^ male mice display abnormal sperm morphology

**Figure 2:**
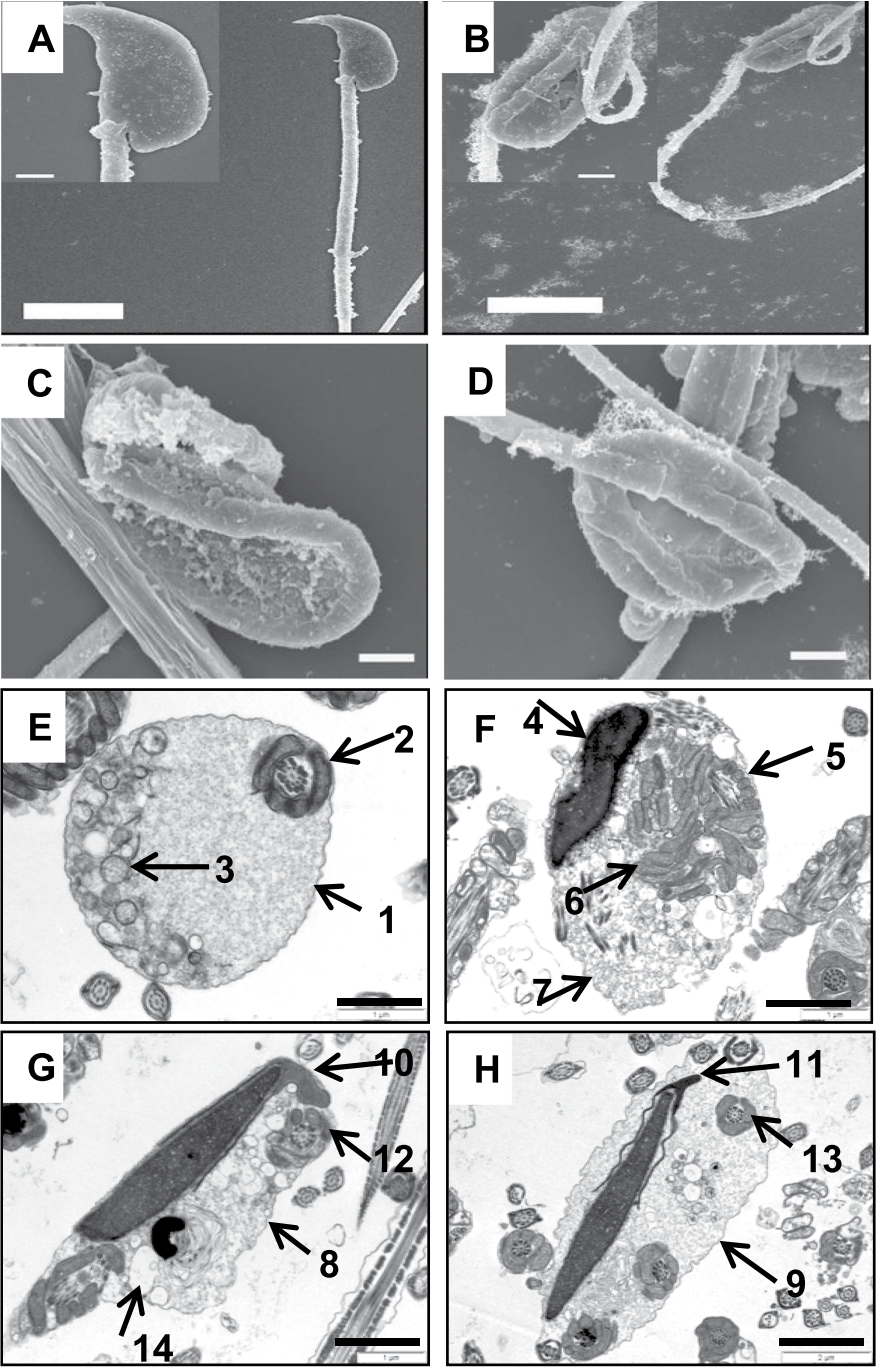
Ultrastructure analyses of WT and *Ncoa5*^+/−^ sperm. (**A**–**D**) Representative SEM analytic images of WT (**A**) and *Ncoa5*^+/−^ epididymal sperm (**B**–**D**). Inserts are the higher magnification images of the head and neck region of the sperm. Scale bars: 5 μm (**A**,**B**); 1 μm (**C**,**D**); inserts: 1 μm. (**E**–**H**) Representative TEM analytic images of WT (**E**) and *Ncoa5*^+/−^ epididymal sperm (**F**–**H**). Scale bars: 1μm. Cross-section of the cytoplasmic droplet (CD) of a WT spermatozoon (**E**). 1: Outer membrane of the CD; 2: middle piece of the sperm containing mitochondrial sheath and axoneme; 3: vacuoles within the CD; Cross-sections of *Ncoa5*^+/−^ bent sperm head and neck region wrapped around by the tail (**F**–**H**). 4: nucleus; 5: middle piece containing mitochondria and axoneme wrapped around the bent head and neck; 6: disrupted mitochondria of the middle piece; 7, 8 and 9: outer membrane of the droplet; 10 and 11: acrosome; 12 and 13: wrapped-around middle piece containing mitochondria and axoneme; 14: large membranous vacuoles.

**Figure 3:**
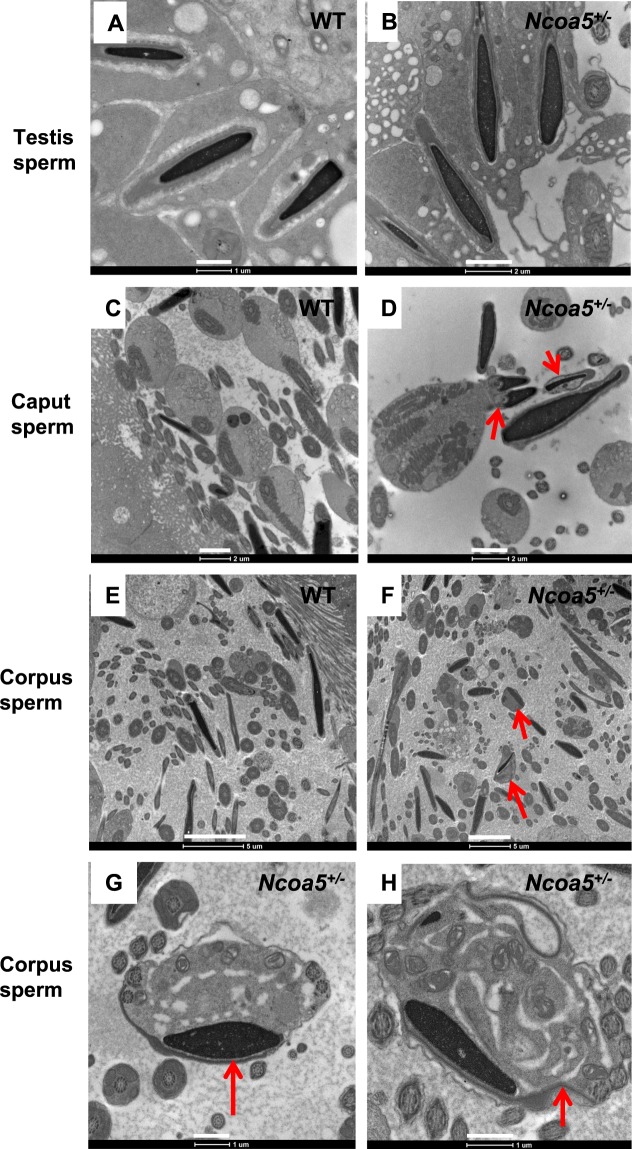
Representative TEM images of *Ncoa5*^+/−^ sperm from different parts of the urinary tract. (**A**,**B**) Representative TEM images of sperm from testis showed comparable morphology between WT (**A**) and *Ncoa5*^+/−^ (**B**), Scale bars (**A**): 1μm, (**B**): 2 μm, (**C**,**D**) TEM images of sperm from WT (**C**) and *Ncoa5*^+/−^ (**D**) caput epididymis. Scale bars (**C**,**D**): 2 μm, (**E**–**H**) TEM images of sperm from WT (**E**) and *Ncoa5*^+/−^ (**F**–**H**) corpus epididymis, lower magnification (**E**,**F**), higher magnification (**G**,**H**). Scale bars (**E**,**F**): 5 μm, (**G**,**H)**: 1μm. Red arrow indicates the abnormal morphology of sperm in *Ncoa5*^+/−^ epididymis.

We next examined the gross anatomy and histology of H & E stained sections of WT and *Ncoa5*^+/−^ testes. WT and *Ncoa5*^+/−^ mice displayed comparable testis size and weight. No significant differences in histology of H & E stained sections were observed between the WT and *Ncoa5*^+/−^ testes (Fig. S2A). However, when the spermatozoa collected from the cauda epididymis were examined under the phase contrast microscope, a significant proportion of spermatozoa from *Ncoa5*^+/−^ mice apparently displayed abnormal morphology compared with *Ncoa5*^+/+^ sperm (Fig. 1E,F). Sybr green and Mito track staining of sperm showed that the heads of many *Ncoa5*^+/−^ sperm were misshapen as compared with WT sperm (Fig. 1E). To uncover structural defects in *Ncoa5*^+/−^ sperm, we compared the ultra-structure of *Ncoa5*^+/−^ sperm with that of WT sperm using scanning electron microscopy (SEM) and transmission electron microscopy (TEM). Under SEM, the heads of some of *Ncoa5*^+/−^ sperm, in contrast to those of *Ncoa5*^*+/+*^ controls (Fig. 2A), appeared to have roughly uneven surface and bend over with the tip of the head toward the tail or to curl like a “golf stick” (Fig. 2B). Some of *Ncoa5*^+/−^ sperm curled as a disk-like structure by wrapping around the head with the neck and middle parts of the tail (Fig. 2B–D). In aid of the observations with light microscopic and SEM, TEM showed that the middle piece consists of disrupted mitochondria and axoneme wrapped around the bent head and neck of *Ncoa5*^+/−^ sperm compared to the head of WT sperm (Fig. 2E–H). In addition, we determined where the sperm developed the abnormal morphology. TEM analysis showed that the morphology of *Ncoa5*^+/−^ sperm collected from testis was comparable to that of WT (Fig. 3A,B). However, *Ncoa5*^+/−^ sperm harvested from caput epididymis had started to exhibit abnormal morphology (Fig. 3C,D). Furthermore, the *Ncoa5*^+/−^ sperm collected from corpus epididymis had severe deformed morphology compared to that of WT sperm (Fig. 3E–H). Therefore, *Ncoa5*^+/−^ sperm appear to acquire the abnormal morphology in the course of the post-gonadal stages of sperm differentiation during transit from the head to the tail of the epididymis, while the morphological structure of epididymis was comparable in *Ncoa5*^+/−^ and *Ncoa5*^+/+^ male mice (Fig. S3).

### Expression of IL-6 in the epithelium was increased in *Ncoa5*^+/−^ epididymis

**Figure 4:**
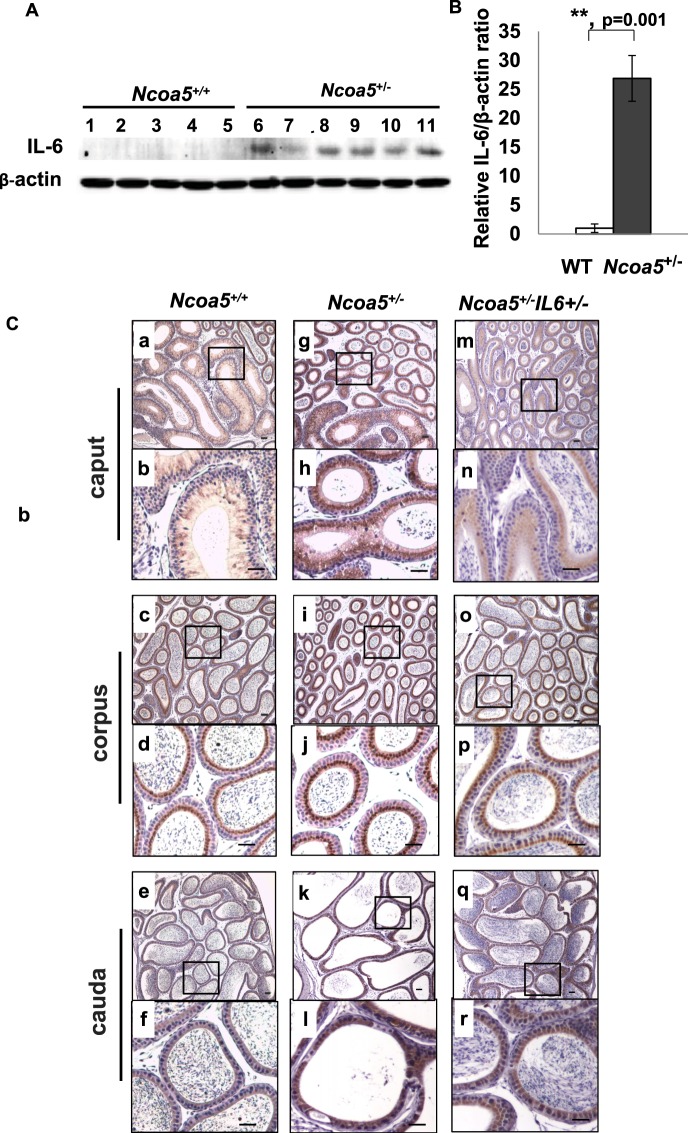
IL-6 expression in epididymis of WT and *Ncoa5*^+/−^ male mice. (**A**) Western blot analysis of IL-6 expression in epididymis of age-matched 13–15 months old WT (n = 5, lanes 1–5) and *Ncoa5*^+/−^ (n = 6, lanes 6–11) male mice using anti-IL-6 and anti-β-actin antibodies. Each lane represents a single tissue lysate of the whole epididymis from a mouse. Cropped blots from the same gel are shown. Uncropped blots are presented in Supplementary Fig. S4. The same blot membrane was stripped and blotted with the same method except without adding the primary antibodies as control (Supplementary Fig. S4C). (**B**) Quantification of data from (**A**). n = the number of mice. **P < 0.01. Error bar ± SEM. (**C**) Representative IL-6 IHC staining of WT, *Ncoa5*^+/−^ and *Ncoa5*^+/−^*Il-6*^+/−^ male mouse epididymides. Immunohistochemical analysis was performed on epididymis from 4.5 months old WT (n = 2), *Ncoa5*^+/−^ (n = 3) and *Ncoa5*^+/−^*Il-6*^+/−^ (n = 3) male mice using an anti-IL-6 antibody. n = the number of mice. Representative IL-6 IHC staining images of WT (a–f), *Ncoa5*^+/−^ (g–l) and *Ncoa5*^+/−^*Il-6*^+/−^ (m–r) epididymal caput (segments 1–2) at lower (a, g and m) and higher (b, h and n) magnification, corpus (segments 7) at lower (c, i and o) and higher (d, j and p) magnification, and cauda (segments 8–9) at lower (e, k and q) and higher (f, l and r) magnification. Black squares specify the areas that are magnified and displayed as high magnification pictures. Scale bars: 50 µm. Immunohistochemical analysis without using IL-6 antibody was performed on epididymis from an *Ncoa5*^+/−^ mouse and a representative image is shown in Supplementary Fig. S5.

We previously reported that the mRNA and protein levels of IL-6 were elevated in the liver of *Ncoa5*^+/−^ male mice and that heterozygous deletion of *Il-6* gene rescued glucose intolerance and hindered HCC development in *Ncoa5*^+/−^ male mice^21^. We therefore compared IL-6 expression in the epididymis between *Ncoa5*^+/+^ and *Ncoa5*^+/−^ male mice using Western blot and immunohistochemistry (IHC) analyses. Expression of IL-6 was significantly increased in epididymis of *Ncoa5*^+/−^ male, compared to epididymis of age-matched *Ncoa5*^+/+^ male mice (Figs 4A,B). IL-6 IHC staining in the epithelium was significantly increased in the caput, corpus and cauda of the epididymis of *Ncoa5*^+/−^ male mice (Fig. 4C,g–l) compared with those of *Ncoa5*^+/+^ male mice (Fig. 4C,a–f), whereas no apparent IL-6 IHC staining was detected in both *Ncoa5*^+/+^ and *Ncoa5*^+/−^ testes (Fig. S5). These results suggest that IL-6 expression is elevated in the epithelial cells of the epididymis in the *Ncoa5*^+/−^ mice.

### Heterozygous deletion of Il-6 improves fertility of *Ncoa5*^+/−^ male mice

**Figure 5:**
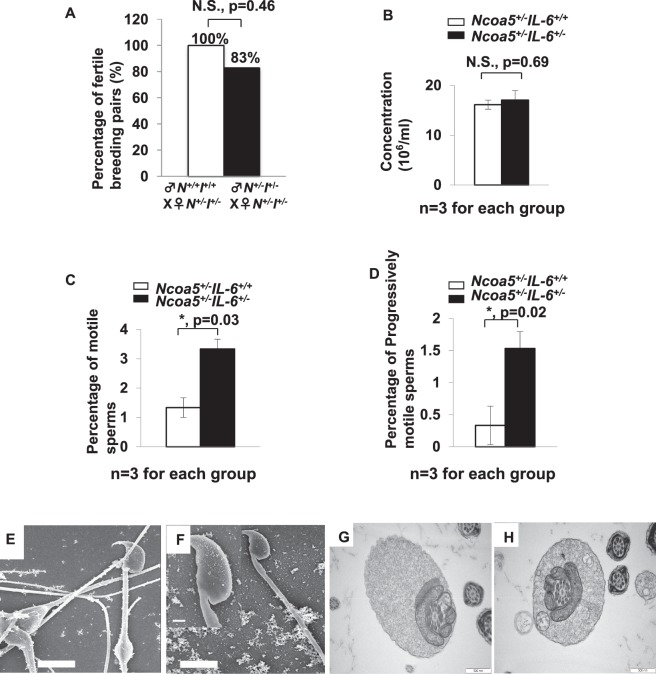
Heterozygous *Il-6* deletion improves fertility and sperm morphology of *Ncoa5*^+/−^ male mice. (**A**) Percentages of fertile *Ncoa5*^*+/+*^*Il-6*^*+/+*^ male mice or *Ncoa5*^+/−^*Il-6*^+/−^ male mice bred with *Ncoa5*^+/−^*Il-6*^+/−^ female mice for six months. White bar, *Ncoa5*^*+/+*^*Il-6*^*+/+*^ male mice (n = 7). Black bar, *Ncoa5*^+/−^*Il-6*^+/−^ male mice (n = 6). n = the number of mice. Two-tailed Fisher’s exact test, N.S.: no significance. (**B**) Epididymal cauda sperm concentrations of 4-month-old *Ncoa5*^+/−^*Il-6*^*+/+*^ and *Ncoa5*^+/−^*Il-6*^+/−^ male mice (n = 3). n = the number of mice. N.S.: no significance. (**C**) Percentage of motile sperm of 4-month-old *Ncoa5*^+/−^*Il-6*^*+/+*^ and *Ncoa5*^+/−^*Il-6*^+/−^ male mice (n = 3). (**D**) Percentage of progressive sperm of 4-month-old *Ncoa5*^+/−^*Il-6*^*+/+*^ and *Ncoa5*^+/−^*Il-6*^+/−^ male mice (n = 3). n = the number of mice. All values are mean ± SEM. *P < 0.05. (**E**,**F**) Representative scanning EM analysis of *Ncoa5*^+/−^*Il-6*^+/−^ epididymal cauda sperm. Insert (**F**) is the higher magnification image of the head and neck region of the sperm. Scale bars: 5 μm; insert: 1 μm. (**G**,**H**) Representative TEM analysis of *Ncoa5*^+/−^*Il-6*^+/−^ epididymal cauda sperm. Scale bars: 500 nm.

**Figure 6:**
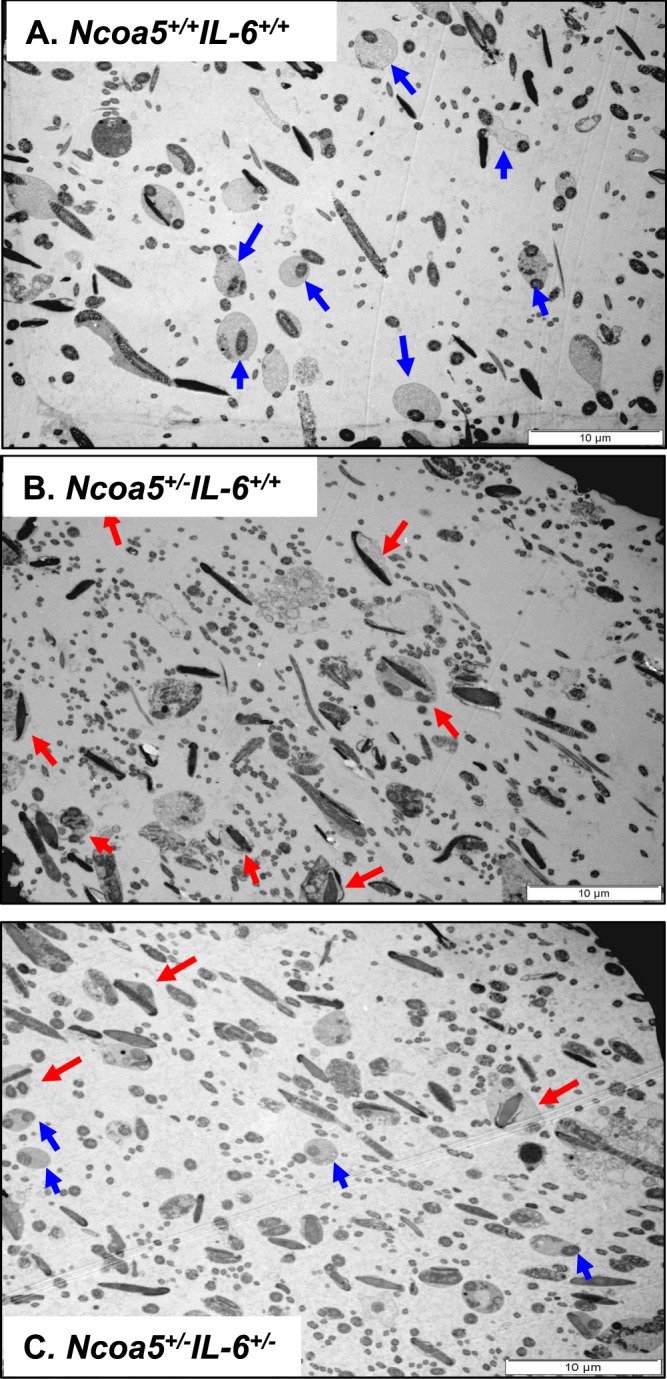
Representative TEM images of corpus epididymal sperm from *Ncoa5*^*+/+*^*Il-6*^*+/+*^, *Ncoa5*^+/−^*Il-6*^*+/+*^ and *Ncoa5*^+/−^*Il-6*^+/−^ male mice. (**A**) TEM of *Ncoa5*^*+/+*^*Il-6*^*+/+*^ corpus epididymal sperm. Blue arrows indicate the normal morphology of the cytoplasmic droplet. (**B**) TEM of *Ncoa5*^+/−^*Il-6*^*+/+*^ corpus epididymal sperm. Red arrows indicate the abnormal morphology of sperm with the head wrapped around by the tail. (**C**) TEM of *Ncoa5*^+/−^*Il-6*^+/−^ corpus epididymal sperm. The presence of both blue (normal) and red (abnormal) arrows indicates the number of normal sperm increases in *Ncoa5*^+/−^*Il-6*^+/−^ male mice. Scale bars: 10 μm.

To determine whether increased IL-6 expression is responsible for the phenotypes observed in *Ncoa5*^+/−^ mice, we generated mice bearing dual heterozygous deletions of *Il-6* and *Ncoa5* genes by crossing *Ncoa5*^+/−^ with *Il-6*^−/−^ (B6.129S6-*Il-6*^*tm1Kopf*^) mice. As expected, IHC staining showed that the epididymal expression of IL-6 was apparently reduced in *Ncoa5*^+/−^ *Il-6*^+/−^ mice (Fig. 4Cm–r). Strikingly, we found that 5 out of 6 *Ncoa5*^+/−^ *Il-6*^+/−^ male mice were able to father litters with 2 to 8 pups per litter when mating with *Ncoa5*^+/−^*Il-6*^+/−^ female mice (Fig. 5A), in contrast to *Ncoa5*^+/−^ male mice shown in Fig. 1A. Analysis of epididymal cauda sperm revealed that sperm from *Ncoa5*^+/−^*Il-6*^+/−^ male mice displayed both increased motility and progressive motility compared to age-matched *Ncoa5*^+/−^*Il-6*^+/+^ male sperm (Fig. 5C,D). As expected, there was no significant difference in the sperm concentration between *Ncoa5*^+/−^*Il-6*^+/−^ and *Ncoa5*^+/−^*Il-6*^+/+^ (Fig. 5B). Consistent with increased and progressive motility, the ultrastructure of sperm from *Ncoa5*^+/−^*Il-6*^+/−^ male mice appeared to be significantly improved as shown by the SEM and TEM scanning analyses (Figs 5E–H and 6). These results suggest that the phenotypes of morphologically abnormal sperm and poor fertility observed in *Ncoa5*^+/−^ male mice were due, at least in part, to IL-6 overexpression in the epididymis.

## Discussion

Genetic abnormalities and/or inflammation in male reproductive organs including epididymitis, orchitis, prostatitis, varicocoele, and testicular torsion as well as drug therapy and lifestyle choices have been known to cause male infertility in human. Nonetheless, it has been reported that 30–75% of infertile male patients are idiopathic^23–25^. It is generally believed that novel inherited and/or acquired mutations in genes involved in spermatogenesis could be the etiological factors in these cases. However, identification of novel inherited genetic abnormalities has been limited because the penetration of such mutations into populations is relatively rare. Herein, using a genetically-engineered mouse model of heterozygous *Ncoa5* deletion, we demonstrated that NCOA5 plays an essential role in male fertility, at least in part through the regulation of IL-6 expression in epididymis. Our results suggest a critical role of NCOA5 in epididymal sperm maturation and further implicate NCOA5 deficiency as a possible etiological risk in human male infertility.

A number of previous studies have indicated roles of inflammatory cytokines including IL-6 in male reproductive function^14^. Although the immune system may be the major source of these cytokines, other cells in the reproductive tract such as epididymal epithelial cells and spermatozoa may also secrete cytokines. Existing evidence has implicated that cytokines including IL-6 can modulate and influence sperm activity and male fertility, as the IL-6 concentration in seminal plasma of infertile men was found to be significantly higher than that of fertile men^26^. Moreover, it was reported that higher level of IL-6 in seminal plasma was negatively correlated with spermatozoa vitality and motility in men^27^. NCOA5 was previously shown to be assembled on the promoter of IL-6 gene and negatively regulate its transcription in hepatic macrophages and heterozygous deletion of *Ncoa5* resulted in increased IL-6 expression in the livers of male mice^21^. Thus, it is possible that NCOA5 may also play an inhibitory role in the regulation of IL-6 expression in epididymal epithelial cells and its inhibition may result in elevated expression of IL-6 in mouse epididymis. In agreement with the previous observations, we showed that IL-6 expression was elevated in the epididymis of *Ncoa5*^+/−^ male mice. Concomitantly, the motility, progression and morphology of the sperm in *Ncoa5*^+/−^ epididymis appeared abnormal. Significantly, we demonstrated for the first time that heterozygous deletion of *Il-6*, partially rescued male mouse infertility phenotype through improving sperm morphology and increasing the sperm motility and progression. This provides *in vivo* evidence to reinforce a causative role of IL-6 overexpression in male infertility. Given the fact that the development of sperm motility and maturation is completed through progressive steps in epididymis^6^ and IL-6 could impact on cell differentiation through the SOCS3/STAT3 signaling pathway^28^, we postulate that elevated IL-6 may contribute to sperm malfunction and infertility of *Ncoa5*^+/−^ male mice through impairing sperm motility and quality. However, it remains to be determined how increased IL-6 expression in epididymis affects development of sperm motility and maturation.

While resulting in this partial improvement of fertility phenotypes, heterozygous deletion of *Il-6* gene was, however, incapable of increasing the activity of *Ncoa5*^+/−^ sperm to the levels comparable to those of WT sperm, suggesting that additional pathogenic effects caused by NCOA5 deficiency may contribute to the development of abnormal sperm in *Ncoa5*^+/−^ male mice. Since *Ncoa5*^+/−^ livers in male mice exhibited chronic inflammation with increased macrophage infiltration and expression of inflammatory cytokines including IL-6 and TNF-α^21^, it is possible that *Ncoa5*^+/−^ epididymis may also develop chronic inflammation with increased expression of multiple inflammatory cytokines. Therefore, we do not rule out the possibility that other cytokines expressed in *Ncoa5*^+/−^ epididymis may also affect the development of sperm in *Ncoa5*^+/−^ male mice. Given the role of NCOA5 in ERα-mediated transcription^18,19^, NCOA5 deficiency might possibly influence expression of other genes regulated by ERα, contributing to the development of infertility. Indeed, significantly reduced levels of proteins such as the sodium/hydrogen exchanger SLC9A3, sodium/bicarbonate cotransporter SLC4A4 and CAR14 in the initial segment of epididymis were observed in ERα knockout (Esr1KO) mice^29–31^. Esr1KO mice are infertile due to significantly reduced sperm motility and progressive motility as well as morphological changes of sperm^29^. Thus, further research is needed to explore whether reduced expression of these three proteins occurs in *Ncoa5*^+/−^ epididymis and also accounts for infertility of *Ncoa5*^+/−^ male mice.

In summary, we have demonstrated that heterozygous deletion of *Ncoa5* resulted in decreased sperm motility and progression and severely impaired fertility in male mice, which were partially rescued by heterozygous deletion of *Il-6* gene. These results suggest NCOA5 as a critical regulator that controls epididymal sperm maturation through regulates IL-6 expression in the epididymis. Our findings not only offer a molecular mechanism underlying male infertility, but also provide a specific target for development of novel therapeutic approaches for human male infertility.

## Materials and Methods

### Mouse mating and reproduction for determination of fertility

Detailed information of generation of *Ncoa5*^+/−^ and *Ncoa5*^+/−^*Il-6*^+/−^ mice was described in a previous publication^21^. All mice were housed in microisolator cages at Michigan State University animal facility. To determine the fertility of male mice, 2-month-old male mouse was housed with age-matched female mouse as monogamous pair and monitored for pup birth for 6 months. Pups in each litter were counted and weaned by 21 days. All experimental procedures on mice were in accordance with the guidelines outlined in the Guide for the Care and Use of Laboratory Animals and approved by the Michigan State University Institutional Animal Care and Use Committee.

### Morphologic analysis

Histological analyses of mouse testis and epididymis were carried out as described previously^21^. Briefly, tissues were dissected and fixed in 10% formalin solution. Fixed tissues were then embedded in paraffin, sectioned and H & E stained by the Histopathology laboratory at Michigan State University. H & E staining was examined under a light microscope (Nikon Eclipse E600) by S.G, H.X., and G.I.P who is a certified veterinarian with extensive experience in mouse reproduction research. For the examination of epididymal sperm, cauda epididymides were collected in M2 medium (Sigma-Aldrich, MO, USA) and immediately transferred into HTF medium (Human Tubal Fluid, EMD Millipore, MA, USA) followed by incubating at 37 °C, 5% CO_2_ for 1 hour. An aliquot of sperm suspension (10 µl) was fixed and examined under a phase contrast microscope (Nikon Eclipse Ti-U inverted microscope).

### Cauda epididymal sperm analysis

Cauda epididymides were harvested from male mice and washed once in M2 medium. Microdrops of HTF medium were prepared at least four hours before, covered with light mineral oil (Chemicon, MilliporeSigma, MA, USA) and incubated at 37 °C, 5% CO_2_. Cauda epididymides were placed individually to microdrops of equilibrated HTF medium, and the sperm was released by gently puncturing the epididymides with a needle under a dissecting scope (Nikon Eclipse Ti-U inverted microscope). Sperm were then capacitated at 37 °C, 5% CO_2_ for 1 hour. Capacitated sperm were mixed with M2 medium for a dilution (1:20), 10 µl of sperm were then added to a slide and analyzed under the Integrated Visual Optical System (IVOS, Hamilton Thorne, Inc, Beverly, MA, USA). About 200 sperm were counted. The concentration, motility and progression of each sperm sample were collected from the IVOS software. Sperm that are highly motile with progressive motility or move fast and progressively are counted as motile or progressively motile respectively.

### Ultrastructural analyses

TEM and SEM analyses were performed according to previously described methods^32^ with minor changes. Specifically, for TEM, the concentration of paraformaldehyde and glutaraldehyde used in our protocol was 2.5%. For dehydration, the blocks were washed with 30%, 50%, 70%, 95% and 100% acetone for 10 min at each concentration. For SEM, CPD 030 Critical Point Dryer (Bal-TEC AG, FL, USA) was used. The EMSCOPE SC500 Sputter coater (Ashford, Kent, Great Britain) was used to coat the sample. Transmitting electron microscope (JEOL100 CXII, JEOL USA, Inc, MA, USA) and scanning electron microscope (JEOL 6610LV, JEOL USA, Inc, MA, USA) were utilized for the electron microscopic analyses and the morphometric images were analyzed by S.G and G.I.P.

### Immunohistochemistry (IHC)

Mouse tissues were fixed in 10% formalin, sectioned in the Histopathology laboratory at Michigan State University. IHC analyses of IL-6 were performed according to the protocol supplied by Cell Signaling Technology (CA, USA).

### Western-blot analysis

Mice were euthanized and then epididymides were harvested and homogenized. Whole protein lysates were subjected to Western-Blot analysis. Western blots were performed and developed by Odyssey infrared scanner according to the manufacturer’s protocols (Li-COR Biosciences, Lincoln, NE, USA) as described previously^33^. Briefly, fluorescent intensity of bands was determined using Odyssey Infrared Imagine System Application Software v3.0, and the expression level for protein of interest was calculated as the ratio of fluorescent intensity between protein of interest and endogenous control protein. NCOA5 (A300–790A 1:1000 dilution), IL-6 (SC-1265, 1:500 dilution) or β-actin (SC-47778, 1:1000 dilution) antibodies were purchased from Bethyl Laboratories (Montgomery, TX, USA) or Santa Cruz Biotechnology (Santa Cruz, CA, USA), respectively. For the control experiment shown in Fig. S4C conducted to confirm the specificity of secondary antibodies, the membrane was firstly stripped with Li-COR NewBlot PVDF Stripping Buffer according to manufacturer’s protocols.

### Statistical analysis

The differences between groups were analyzed using unpaired Student’s 2-tailed t test or two-tailed Fisher’s exact test. n = the number of mice per group. Values are expressed as Mean ± SEM. P < 0.05 is considered statistically significant.

## Supplementary information

Supplemental Information
