# Supplementary material for: NCOA5 Haplo-insufficiency Results in Male Mouse Infertility through Increased IL-6 Expression in the Epididymis
Source: Sci Rep. 2019 Oct 29;9:15525. doi: 10.1038/s41598-019-52105-9 (PMC6820533; doi:10.1038/s41598-019-52105-9)
Supplement: Supplementary file 1 — Supplemental Information [file 41598_2019_52105_MOESM1_ESM.pdf]

Supplemental Information

NCOA5 Haplo-insufficiency Results in Male Mouse Infertility through Increased IL-6 Expression in the Epididymis

Shenglan Gao, Yueqi Zhang, Chengfeng Yang, Gloria I. Perez, and Hua Xiao

Figure S1

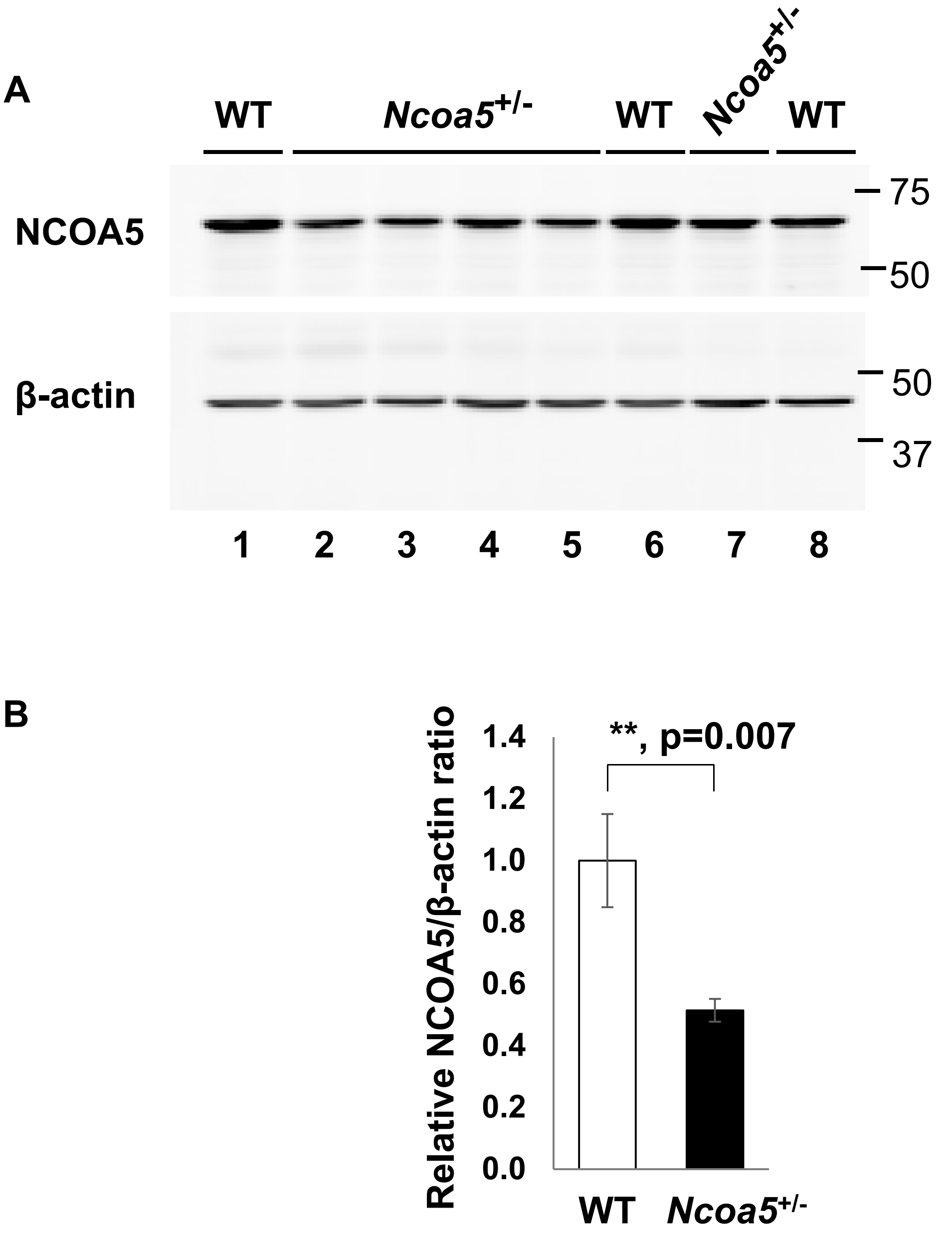

Figure S1 *Ncoa5* expression level in WT and *Ncoa5*$^{+/-}$ male mouse testis. (A) Western-blot analysis of *Ncoa5* expression in testis of age-matched 5 months old WT (n=3, lanes 1, 6, 8) and *Ncoa5+/-* (n=5, lanes 2-5, 7) male mice. Cropped blots are shown. Uncropped blots are shown in Supplementary Figure S4. (B) Quantification of data from (A). Unpaired two-tailed t-test, **p≤0.01. Error bar ± SEM.

Figure S2

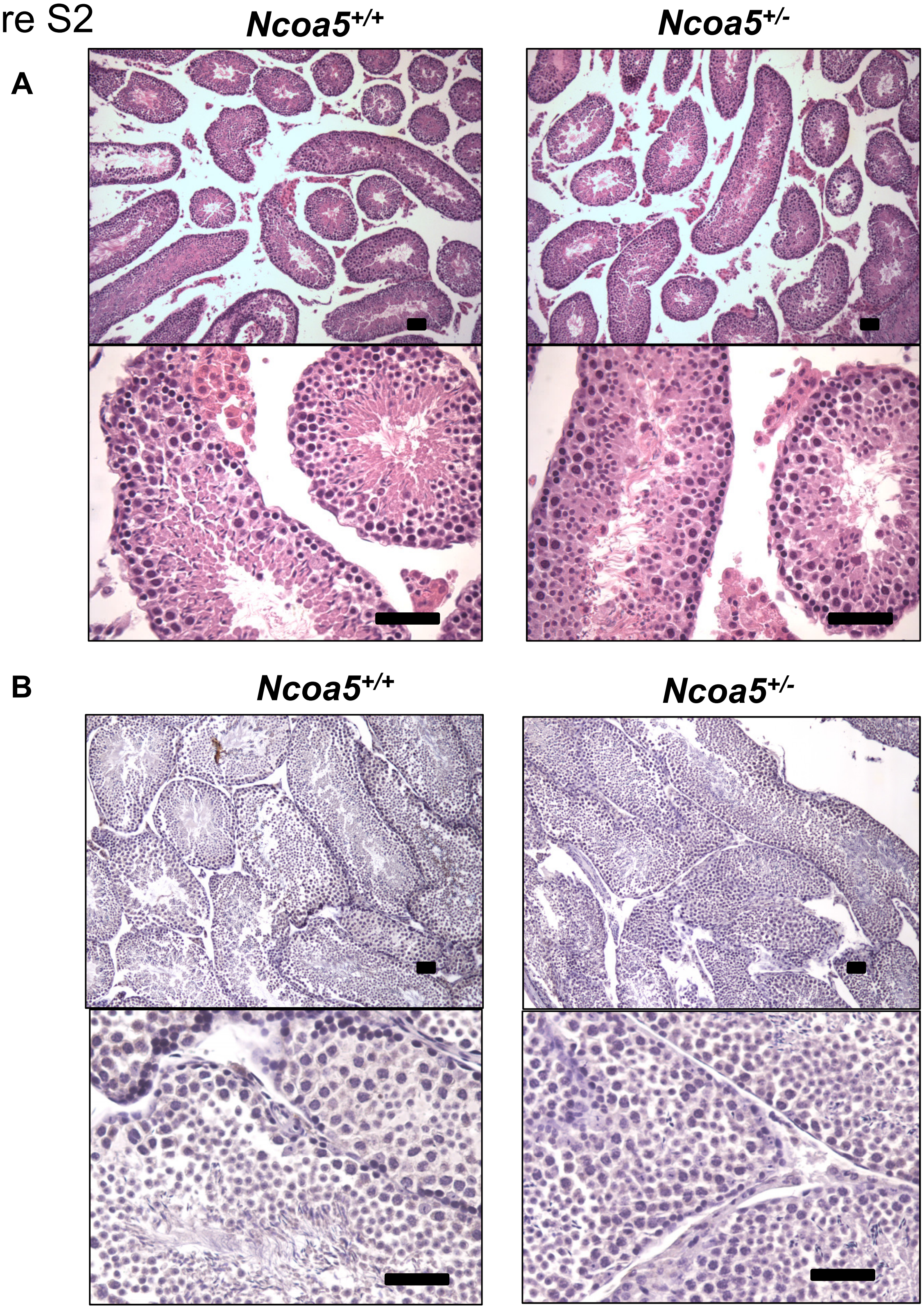

Figure S2 Testicular morphology and IL-6 staining of WT and *Ncoa5*$^{+/-}$ male mice. (A) Representative H&E stained testis of *Ncoa5*$^{+/+}$ (left) and *Ncoa5*$^{+/-}$ (right) mice at lower (upper) and higher magnification (lower). Scale bar: 50 µm. (B) Representative IL-6 IHC analysis of testis from *Ncoa5*$^{+/+}$ (left) and *Ncoa5*$^{+/-}$ (right) mice at lower (upper) and higher magnification (lower). Scale bar: 50 µm.

Figure S3

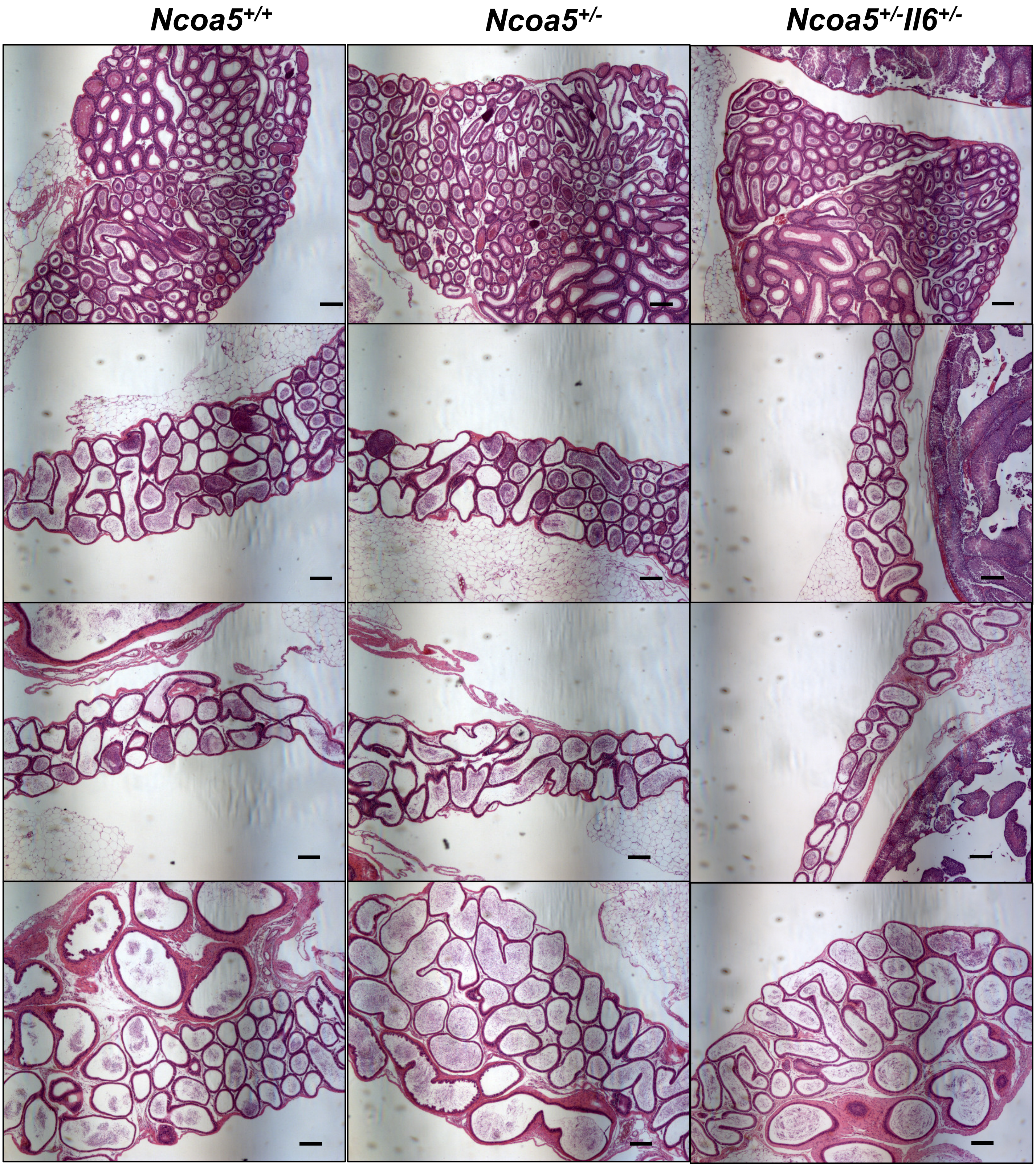

Figure S3 Epididymal morphology of WT, *Ncoa5*$^{+/-}$ and *Ncoa5*$^{+/-}$*Il6*$^{+/-}$ male mice. Representative images for H&E stained whole epididymis of *Ncoa5*$^{+/+}$ (left), *Ncoa5*$^{+/-}$ (middle) and *Ncoa5*$^{+/-}$*Il6*$^{+/-}$ (right) mice are shown in different segments. Top to bottom: caput to cauda of one epididymis. Scale bar: 200 μm.

Figure S4

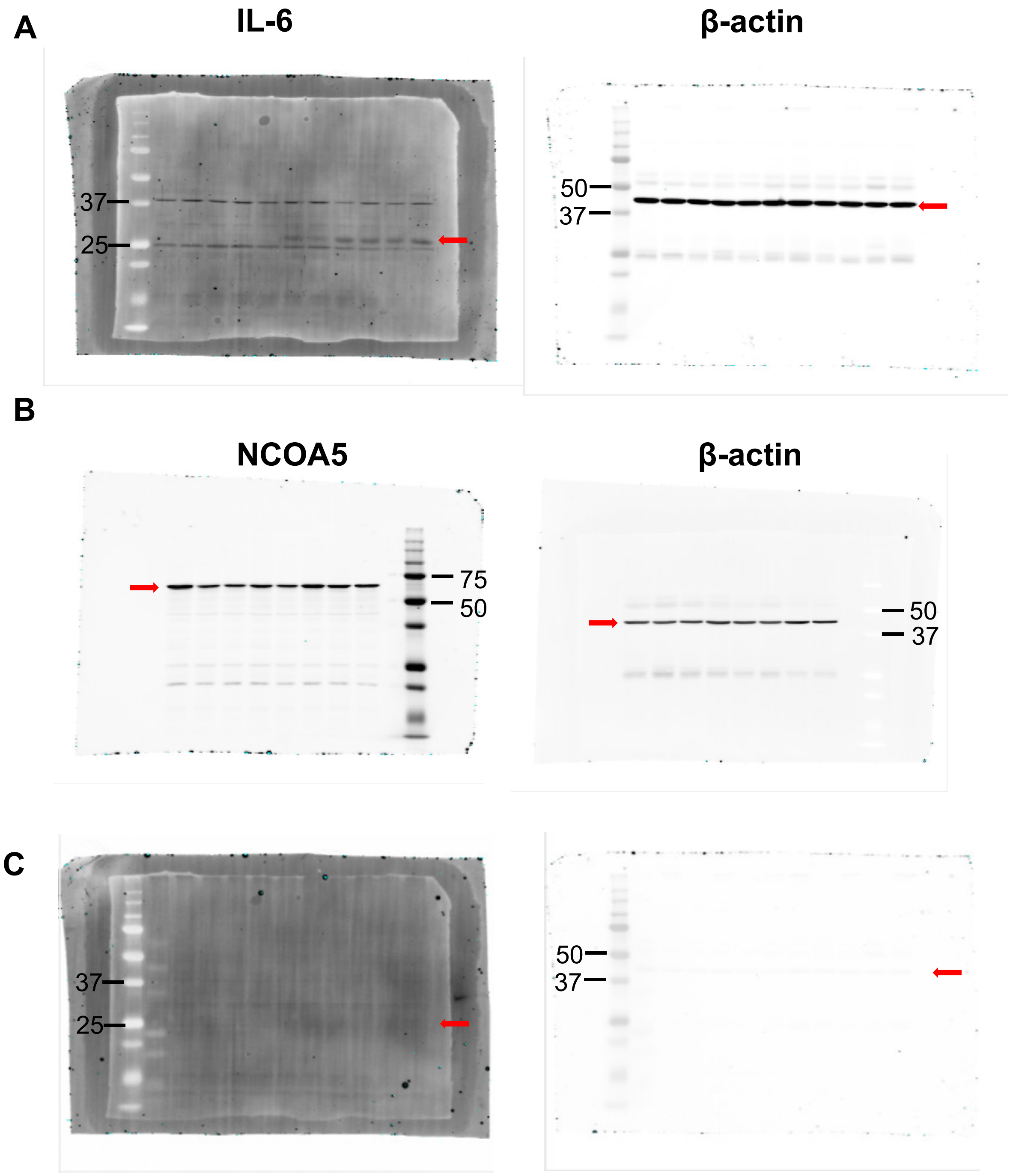

Figure S4 Full-length western blots. (A) Full-length western blots of Figure 3. (B) Full-length western blots of Supplementary Figure S1. (C) Full-length western blot without primary antibody for IL-6 and β-actin 3 to show that the displayed bands for IL-6 and β-actin were not a result of non-specific binding of the secondary antibodies. The same membrane from Figure 3 was stripped using LICOR NewBlot PVDF Stripping Buffer and incubated with the same secondary antibodies as Figure. Photos were captured with the exact same settings. Left and right figures show the fluorescent channels for secondary antibodies for IL-6 or β-actin, repectively.

Figure S5

Ncoa5$^{+/-}$

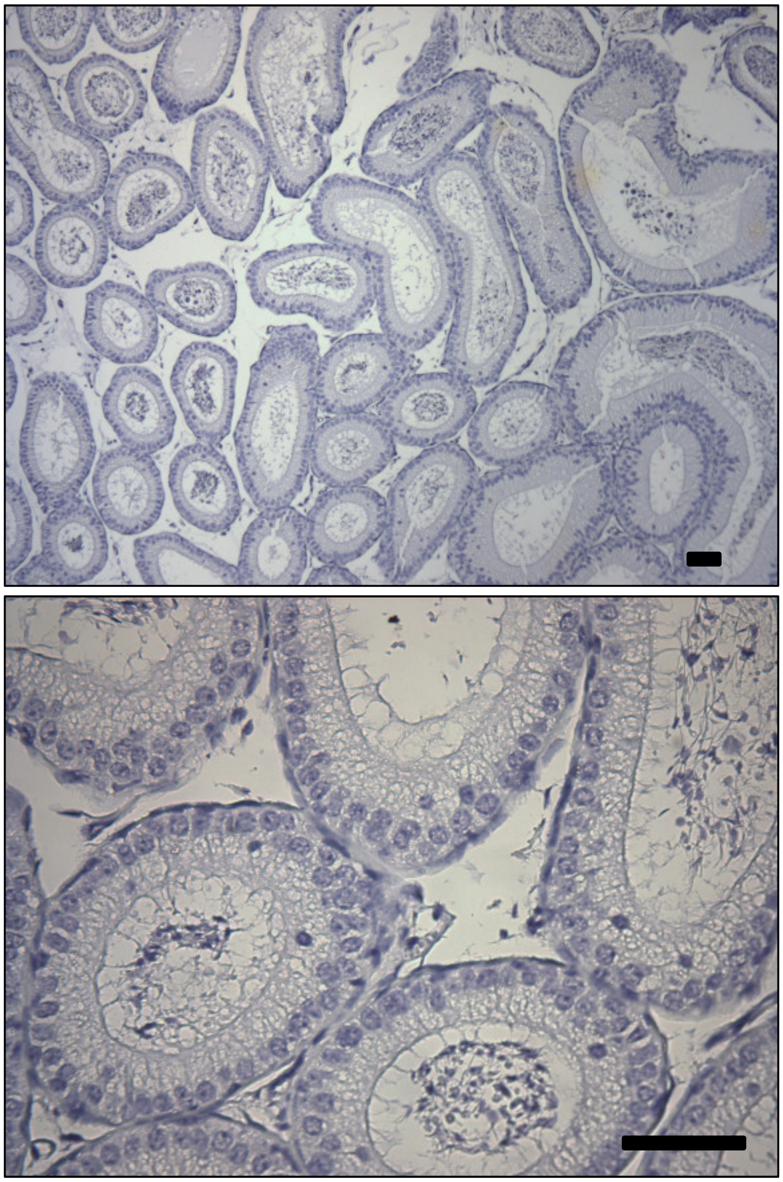

Figure S5 Technological control for IL-6 immunohistochemical staining of epididymis. Control immunohistochemical staining without IL-6 primary antibody. Representative images of caput epididymis of *Ncoa5*$^{+/-}$ mice at lower (top) and higher magnification (bottom). Scale bar: 50 μm.
